# The potential of self- supervised learning in embryo selection for IVF success

**DOI:** 10.1016/j.patter.2024.101012

**Published:** 2024-07-12

**Authors:** Guanqiao Shan, Yu Sun

**Affiliations:** 1 Department of Mechanical and Industrial Engineering, University of Toronto, Toronto, ON M5S 3G8, Canada; 2 Department of Computer Science, University of Toronto, Toronto, ON M5S 2E4, Canada

## Abstract

How to select the “best” embryo for transfer is a long-standing question in clinical *in vitro* fertilization (IVF). Wang et al. proposed a multi-modal self-supervised learning framework for human embryo selection with a high accuracy and generalization ability.

## Main text

Since the birth of the first *in vitro* fertilization (IVF) child in 1978, more than 10 million children have been born with IVF treatment worldwide. However, IVF success rate has remained relatively unchanged over the last 20 years.^1^ Embryo selection is a critical step to select the “best” embryo(s) for transfer, but how to determine the selection strategy remains a challenge in IVF practice.

Clinically, manual morphological grading is universally used for embryo selection. Embryologists visually observe the embryo morphology at specific time points (e.g., day 3, day 5, or day 6) before embryo transfer. Although an international consensus was established in 2011, manual grading suffers from large intra-/inter-observer variability due to the qualitative grading criteria and inherent subjectivity.^2^ With the emergence of time-lapse imaging, time-lapse videos have also been used to assess embryo development patterns, known as morphokinetics, for embryo selection. Currently, disparities still exist in the selection of morphokinetic events, and criteria in consideration of multiple morphokinetic events remain unestablished.^3^ Additional techniques such as preimplantation genetic testing (PGT) have been employed for genetic screening and diagnosis of embryos. Due to the invasive nature of the biopsy procedure^4^ and the financial burden for genetic testing, whether PGT should be routinely used in IVF treatment is still under debate.

Artificial intelligence (AI) has gained significant traction for non-invasive embryo selection in recent years. Various machine learning models have been investigated. These models use different features as input, including embryo images, time-lapse videos, and/or clinical features. The quality of an embryo is assessed based on the model’s output probability in terms of euploidy, pregnancy, or live birth. These machine learning models aimed to mitigate human subjectivity and demonstrated improved performance in embryo selection compared to embryologists’ manual assessment.^5^ Existing models use supervised learning frameworks that require the ground truth labels of clinical outcomes for each embryo. Hence, embryos not enrolled in PGT cycles or not selected for transfer are not seen by these models. It is questionable whether a model trained on a biased dataset can well generalize to different populations. Additionally, since only a small fraction of embryos is transferred and results in subsequent clinical outcomes, it is challenging to utilize this limited number of embryos for tuning a machine learning model with millions of parameters.

**Figure 1:**
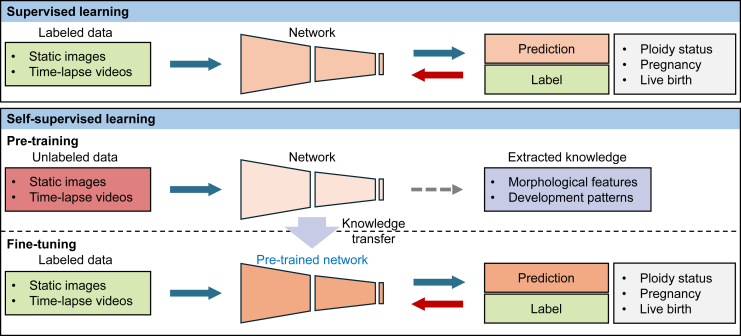
Supervised learning vs. self-supervised learning for embryo selection

Different from supervised learning frameworks, self-supervised learning automatically generates supervisory labels for unlabeled data by exploring the data features and relationships within the dataset, which is termed pre-training. Then a small dataset with labels is used to fine-tune the pre-trained model (see Figure 1). For embryo selection, the available unlabeled data for self-supervised learning can be orders of magnitude more than the labeled data for supervised learning. The large amount of data can help alleviate dataset bias and enable the model to recognize more subtle features for improving its performance in embryo selection. Kragh et al.^6^ demonstrated that their self-supervised learning model fine-tuned with 16% of the labeled dataset outperformed the supervised learning model trained by the entire labeled dataset on pregnancy prediction when using time-lapse videos of human embryos. They showed that the pre-trained model was able to cluster embryos with similar development patterns and the subsequent fine-tuning process effectively correlated these patterns with the pregnancy likelihood.

Recently, Wang et al.^7^ proposed a generalized self-supervised learning framework for human embryo selection. The model was pre-trained on unlabeled multi-modal data, including images and time-lapse videos from day 3, day 5, and day 6 embryos. Visual and temporal encoders were used to collaboratively extract spatial and temporal features from input embryo images and videos. The authors demonstrated that the pre-trained model was able to cluster embryos with similar morphological features as well as development patterns. Then the pre-trained model was fine-tuned by embryo images with manual grading labels and live birth outcomes and by time-lapse videos with PGT results for aneuploidy. The size of the dataset with labels used for fine-tuning was ∼10% of that used for pre-training (i.e., ∼2,000 vs. ∼20,000 embryos). Although fine-tuned by a small, labeled dataset, external validations showed a strong generalization ability of the self-supervised learning model among different data sources.

The generalization ability of machine learning models for embryo selection has been a long-standing issue mainly caused by the limited quantity of clinical data and the inevitable data bias.^8^ The study by Wang et al.^7^ illustrates the potential of self-supervised learning in improving the generalization ability for embryo selection. First, self-supervised learning extracts key knowledge from unlabeled data and thus requires less quantity of labeled data. With the advancement of imaging technology and laboratory standardization, tens of thousands of embryo images and time-lapse videos are recorded globally on a daily basis. Data from different IVF centers may be used together for pre-training regardless of the different labeling criteria among them, for instance, different morphological grading criteria (ASEBIR vs. Gardner system)^9^ or different genetic testing platforms (FISH vs. NGS). Although a large amount of embryo data is recorded, only a small fraction has clinical outcomes. Unlike supervised learning, self-supervised learning does not require manual allocation of pseudo labels to those embryos without clinical outcomes, for instance, labeling non-transferred embryos as negative results. Instead, it directly learns from the unlabeled embryo data to avoid introducing additional noise.

Second, self-supervised learning may alleviate data bias. In IVF treatment, not all patients enroll in PGT cycles, and only a small fraction of embryos are transferred. Existing supervised learning models were all built on biased clinical datasets. On the contrary, self-supervised learning is capable of acquiring generalized knowledge from all types of patients and embryos via pre-training. The knowledge is then used in the fine-tuning process to distinguish the subset of embryos with clinical outcomes. Therefore, although the dataset used for fine-tuning is biased, self-supervised learning can still maintain strong generalization ability, as demonstrated by the external validation results in Wang’s study.^7^

Finally, self-supervised learning is able to handle datasets with noisy labels since the learning framework is not completely constrained by labeled data. Embryo data with manual grading labels can degrade the supervised machine learning models by intra-/inter-observer variability, leading to limited performance in automatic embryo grading. Embryo data with clinical outcomes, such as pregnancy or live birth labels, are coupled with maternal characteristics that may distract the models from the embryo itself. In the pre-training process, self-supervised learning clusters embryos only based on the similarities of their morphological features and development patterns without human interference or other external factors. Thus, it can minimize the adverse effect from the noisy labels and has the potential to maintain a relatively high accuracy trained by datasets with noisy labels.

Self-supervised learning provides a promising solution for embryo selection considering the nature of clinical embryo data. It demonstrates a high accuracy and strong generalization ability for embryo grading, non-euploidy prediction, and live birth prediction. However, clinical interpretation of these models is still underexplored. The limited interpretability raises epistemic and ethical concerns that may impede their application in clinical IVF. Furthermore, large and comprehensive randomized controlled trials (RCTs) are still lacking. These RCTs are essential to confirm the clinical value of these machine learning models, in which diverse populations must be enrolled to assess their generalization ability.
